# Glenoid baseplate fixation using hybrid configurations of locked and unlocked peripheral screws

**DOI:** 10.1007/s10195-016-0438-3

**Published:** 2017-01-11

**Authors:** Nathan T. Formaini, Nathan G. Everding, Jonathan C. Levy, Brandon G. Santoni, Aniruddh N. Nayak, Cooper Wilson

**Affiliations:** 1 Holy Cross Orthopedic Institute, 5597 North Dixie Highway, Fort Lauderdale, FL 33334 USA; 2 Phillip Spiegel Orthopaedic Research Laboratory, Foundation for Orthopaedic Research and Education, Tampa, USA; 3 Department of Orthopaedics and Sports Medicine, University of South Florida, Tampa, USA

**Keywords:** Reverse shoulder arthroplasty, Glenosphere, Glenoid baseplate, Hybrid screw fixation, Micromotion

## Abstract

**Background:**

The use of peripheral locked screws has reduced glenoid baseplate failure rates in reverse shoulder arthroplasty. However, situations may arise when one or more non-locked screws may be preferred. We aimed to determine if different combinations of locked and non-locked screws significantly alter acute glenoid baseplate fixation in a laboratory setting.

**Materials and methods:**

Twenty-eight polyurethane trabecular bone surrogates were instrumented with a center screw-type glenoid baseplate and fixated with various combinations of peripheral locked and non-locked screws (1-, 2-, 3- and 4-locked con). Each construct was tested through a 55° arc of abduction motion generating compressive and shear forces across the glenosphere. Baseplate micromotion (μm) was recorded throughout 10,000 cycles for each model.

**Results:**

All constructs survived 10,000 cycles of loading without catastrophic failure. One test construct in the 1-locked fixation group exhibited a measured micromotion >150 μm (177.6 μm). At baseline (*p* > 0.662) and following 10,000 cycles (*p* > 0.665), no differences were observed in baseplate micromotion for screw combinations that included one, two, three and four peripheral locked screws. The maximum difference in measured micromotion between the extremes of groups (1-locked and 4-locked) was 29 µm.

**Conclusions:**

Hybrid peripheral screw fixation using combinations of locked and non-locked screws provides secure glenoid baseplate fixation using a polyurethane bone substitute model. Using a glenosphere with a 10-mm lateralized center of rotation, hybrid baseplate fixation maintains micromotion below the necessary threshold for bony ingrowth.

**Level of Evidence:**

N/A/, basic science investigation.

## Introduction

Improvements in glenoid baseplate fixation have been critical to the success of the modern reverse shoulder replacement. Recognition of the importance of baseplate tilt [1–3], optimal screw placement [4–9], glenoid reaming techniques [10], peripheral screw fixation [11], baseplate shape and position [9, 12, 13], and introduction of bone ingrowth technologies [13] have contributed to avoiding the catastrophic baseplate failures described using first-generation reverse shoulder replacements [14–21]. In combination with improved surgical technique and implant enhancements, baseplate failures are less common [6].

One key modification which helped improve baseplate fixation was the introduction of peripheral locked screws. First tested by Harman et al. [11], locked screw use significantly enhanced baseplate fixation and minimized micromotion at the baseplate−glenoid bone interface. This was also observed in clinical practice. Frankle et al. initially reported an 11% baseplate failure rate using peripheral non-locked 3.5-mm screws [22]. However, in a 5-year follow-up series using the same implant with 5.0-mm peripheral locked screws, the baseplate failure rate was reduced to 0% [23]. This has led most surgeons to utilize locked screws for nearly all peripheral screw fixation opportunities when using a central screw-based baseplate.

There are, however, clinical scenarios when a surgeon may prefer to use non-locked peripheral screws. The concept of hybrid fixation, where combinations of locked and non-locked screws are used together, has been a longstanding feature used in locked plate osteosynthesis and has been shown to be biomechanically similar while also providing the potential benefits of compression or aiding with reduction [24–28]. Variations in hybrid peripheral screw combinations have not been tested for reverse shoulder glenoid baseplate fixation. The purpose of this study was to evaluate initial glenoid baseplate fixation using a variety of combinations of locked and non-locked peripheral screws. We hypothesized that glenoid baseplate micromotion would be sufficiently minimized using all combinations of locked and non-locked peripheral screws.

## Materials and methods

Twenty-eight synthetic trabecular bone surrogate cylinders (Sawbones^®^ Model #1522-12, Rigid Cellular Foam; Pacific Research Laboratories, Vashon, WA, USA) with a nominal density of 0.32 g/cm^3^ (ASTM F1839-08) were utilized in the current investigation. This nominal was chosen as an intermediary between poor quality (0.24 g/cm^3^) and good quality (0.48 g/cm^3^) cancellous bone used by others in studies of similar scope [33]. Upon receipt of the blocks (40 × 130 × 180 mm) each was machined into cylinders measuring 44.5 mm in diameter and 40 mm in height.

This study used the glenoid components of a single reverse shoulder arthroplasty system (RSP^®^; DJO Global, Austin, TX, USA). The baseplate consists of a single central 6.5-mm lag screw in combination with four peripheral screw holes, which can be used in locked or non-locked fashion. The underside of the baseplate is coated with hydroxyapatite to facilitate bony ingrowth in vivo. All baseplates were fixed perpendicular to the bone block substitutes. Briefly, a 2.5-mm drill was used to define the trajectory for the baseplate’s central screw. A 6.5-mm tap was used to create the threads for the central screw. With the tap still in place, a reamer was used to create the circumferential concavity required to accommodate the underside of the baseplate, which was then inserted and tightened to a maximum torque of 3.5 N-m (Model DID-04 Digital Torque Screwdriver; Imada, Inc. Northbrook, IL, USA). For this study, we evaluated baseplate fixation using four combinations of locked (5-mm diameter) and (3.5-mm diameter) non-locked peripheral screws in seven (*n* = 7) cylinders per fixation group—(1) 1 locked screw + 3 non-locked screws (1-locked), (2) 2 locked screws + 2 non-locked screws (2-locked), (3) 3 locked screws + 1 non-locked screw (3-locked), and (4) 4 locked screws (4-locked). All locked and non-locked screws were the same length as determined by a retrospective analysis of a consecutive series of 100 reverse shoulder arthroplasty surgeries using the same glenoid baseplate. This identified an average peripheral screw length of 21 mm. Thus, 22-mm screws were selected for all locked and non-locked screws.

**Fig. 1:**
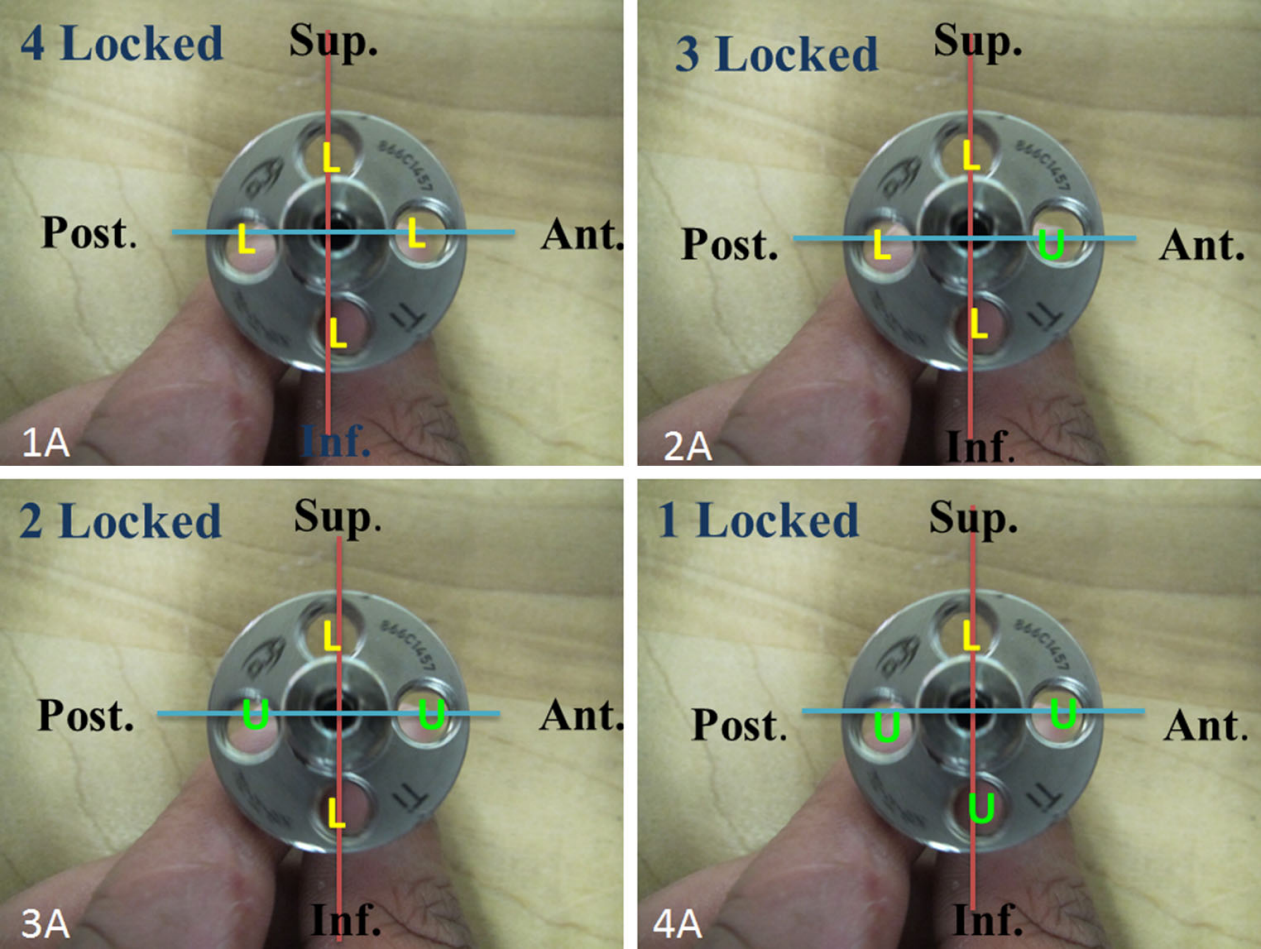
Position of locked and unlocked screws within the baseplate for each configuration.* 1a* Baseplate with four locked screws,* 2a* baseplate with three locked and one unlocked screw,* 3a* baseplate with two locked and two unlocked screws,* 4a* baseplate with one locked and three unlocked screws. *L* locked, *U* unlocked

**Fig. 2:**
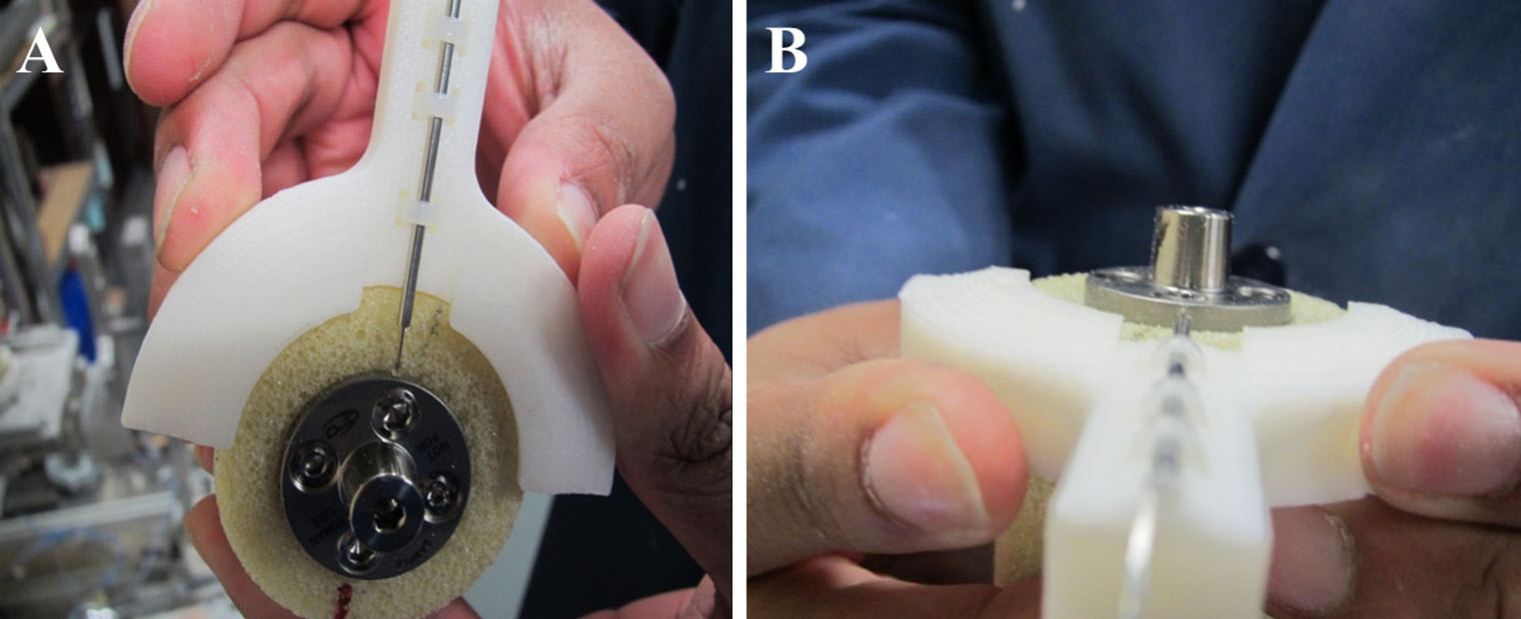
The differential variable reluctance transducer (DVRT) placed in direct contact with the inferior aspect of the baseplate

For peripheral screw placement, a 4-in-1 drill guide was used which allows for drilling and placement of screws (after the inner sleeve is removed) in a constant perpendicular orientation to the baseplate. For non-locked and locked peripheral screws, respectively, 2.5 and 4-mm drills were used to define the screw trajectory in the cylinder. On the circular surface of the baseplate-instrumented Sawbones^®^ cylinder, one screw hole was arbitrarily defined as superior, with anterior, inferior and posterior orientations defined thereafter in clockwise fashion, thereby mimicking a shoulder’s right-sided glenoid orientation (Fig. 1). For the 1-locked screw configuration, the locked peripheral screw was placed in the superior screw hole. In the 2-locked group, locked screws were placed superiorly and inferiorly. Finally, for the 3-locked screw configuration, locked screws were placed superiorly, inferiorly, and posteriorly. Peripheral non-locked screws were advanced to a depth of 22 mm in the foam cylinder and tightened to 0.7 N-m using a torque limiting screw driver. This torque magnitude was determined in pilot experiments and represents the highest, consistently achievable torque in this particular Sawbones^®^ block density without loss of non-locked screw purchase or applied torque. All locked screws were tightened to 2.5 N-m with care taken not to cross the threads. Thereafter, a differential variable reluctance transducer (DVRT) (Model MG-DVRT^®^; LORD MicroStrain^®^ Sensing Systems, Williston, VT, USA) with 3 μm accuracy, was mounted to the instrumented surface of the Sawbones^®^ in the inferior/superior direction with a custom-designed attachment rig such that the sensor was in contact with the periphery of the baseplate immediately adjacent to the superior screw hole (Fig. 2). Inferior/superior micromotion was of primary interest given the orientation of the applied loads in our test setup, which were applied to the baseplate in the scapular plane to simulate abduction and the primary function of reverse shoulders. Out-of-plane micromotion in the anterior/posterior direction was not measured because no shear loads were applied in the axial plane. After screw placement and DVRT attachment, a 32-mm neutral glenosphere (DJO Global, Austin, TX, USA) was impacted onto the baseplate. This glenosphere has a center of rotation 10 mm from the glenoid surface.

**Fig. 3:**
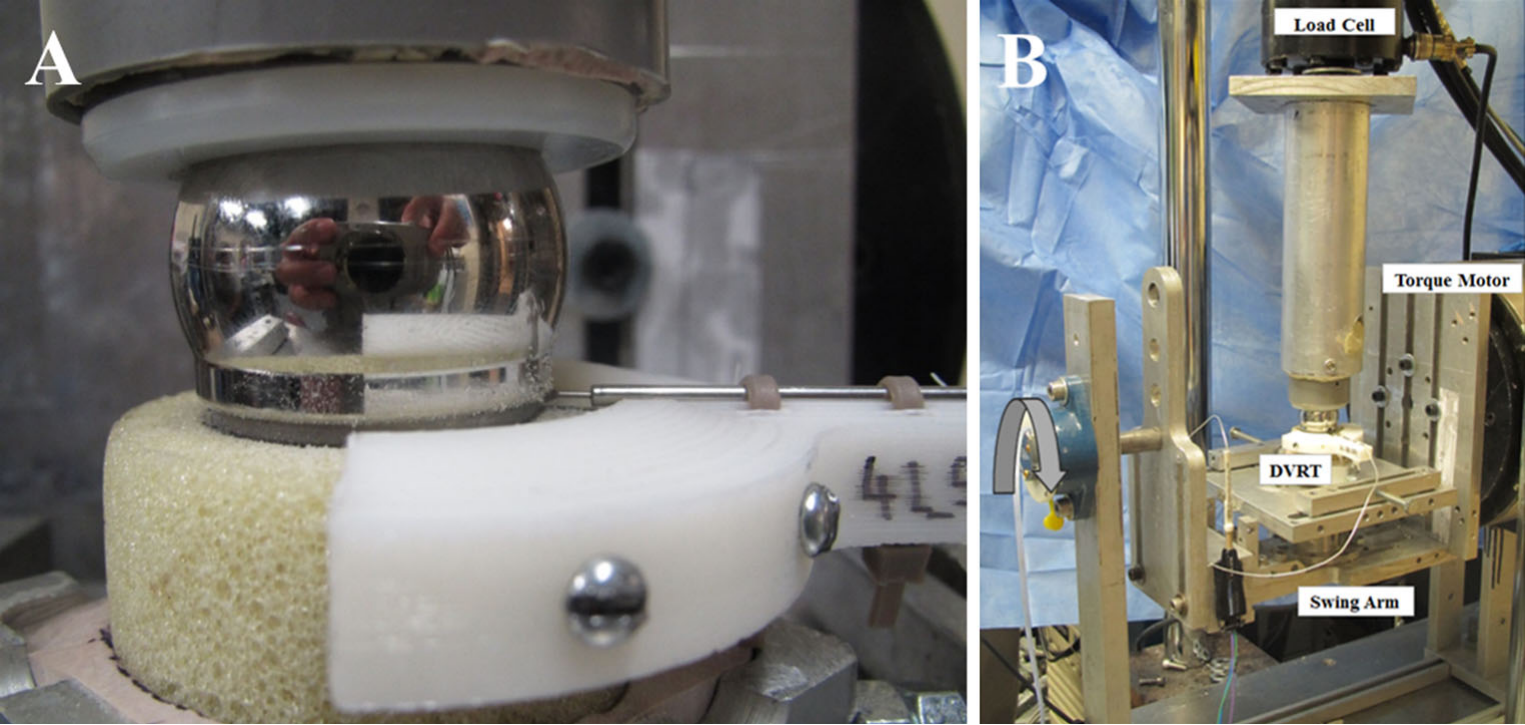
Mechanical testing setup. **a** Close-up photograph detailing the test block with implanted baseplate and 32 neutral glenosphere loaded through a neutral polyethylene liner. **b** Photograph detailing testing set up with swing arm, load cell, torque motor, and DVRT

**Fig. 4:**
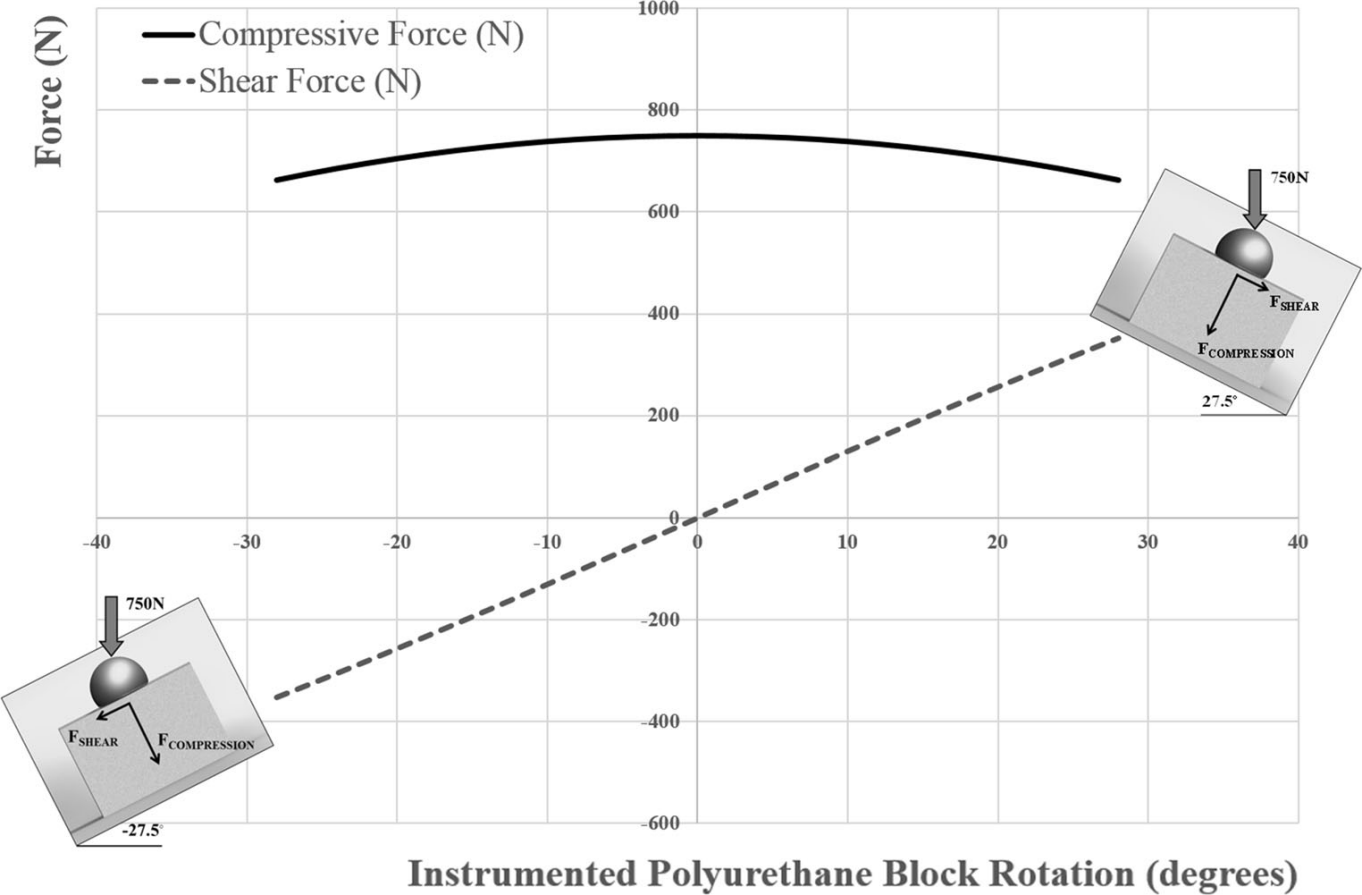
Shear and compressive forces throughout of the 55º arc of simulated abduction motion

We adopted a testing methodology identical to our previously published work [37]. Briefly, the instrumented Sawbones^®^ cylinder was mounted in a swing arm attached to the torque motor of a servoelectric test frame (Model 800LE; Test Resources, Shakopee, MN, USA). Test specimens were cycled through a 55° arc of motion at 1 Hz, while the test frame’s actuator applied a constant 750 N compressive force via a glenosphere size-matched humeral component polyethylene neutral liner, in similar fashion to the methods adopted by previous authors (Fig. 3) [13, 29, 37]. The orientation of the test block was such that the arc of motion occurred in the scapular plane defined by the superior and inferior screw holes of the baseplate, thus serving to approximate a 25°−80° humeral abduction motion relative to a fixed scapula. This loading setup induces a maximum shear force of 346 N at the extremes of the motion arc from neutral (i.e., ±27.5°) and a 750 N compressive load at the neutral position (Fig. 4). The continuous analog voltage output from the DVRT was queried at the beginning of testing and every 500 cycles thereafter through 10,000 cycles of loading. The measured voltage was related to baseplate micromotion (μm) using the manufacturer’s provided linear calibration coefficient. Here, micromotion was defined as the average of the absolute values of micromotion measured by the DVRT at the arc of motion extremes (+27.5° and −27.5°) over 20 loading cycles for each data collection time point. Testing was stopped if catastrophic construct failure was grossly observed.

Micromotion data were analyzed using a 2-way ANOVA for intra-group (time) and inter-group (fixation) comparisons and post hoc comparisons were performed with Bonferroni correction at a significance level of 0.05. The number of test constructs in which micromotion was measured >150 μm threshold commonly accepted for osseous integration was reported.

## Results

All 28 instrumented cylinders survived 10,000 cycles of loading without catastrophic failure. No gross evidence of baseplate subsidence into the test blocks was noted in any specimen. One test construct in the 1-locked fixation group exhibited a measured micromotion >150 μm (177.6 μm) (Table 1). This was observed after 9500 cycles. No constructs in the 2-locked, 3-locked or 4-locked fixation groups exhibited micromotion >150 μm.

**Table 1:** Measured micromotion (μm) at baseline and at 10,000 cycles for each test specimen

Test block	Hybrid screw fixation group							
	1-locked		2-locked		3-locked		4-locked	
	Baseline	10,000 cycles	Baseline	10,000 cycles	Baseline	10,000 cycles	Baseline	10,000 cycles
1	56.2	123.7	58.8	143.1	33.6	39.5	55.8	68.1
2	36.9	40.1	37.0	54.2	51.1	71.3	49.0	78.6
3	68.8	89.1	40.3	43.1	42.4	49.9	42.2	53.7
4	70.2	124.7	51.5	84.5	61.4	121.6	48.5	91.0
5	53.7	177.6	45.7	74.4	62.4	96.8	56.9	73.7
6	52.1	61.0	47.7	89.7	37.8	49.1	38.5	45.2
7	49.9	68.7	47.4	47.6	43.5	78.6	38.4	66.5

**Fig. 5:**
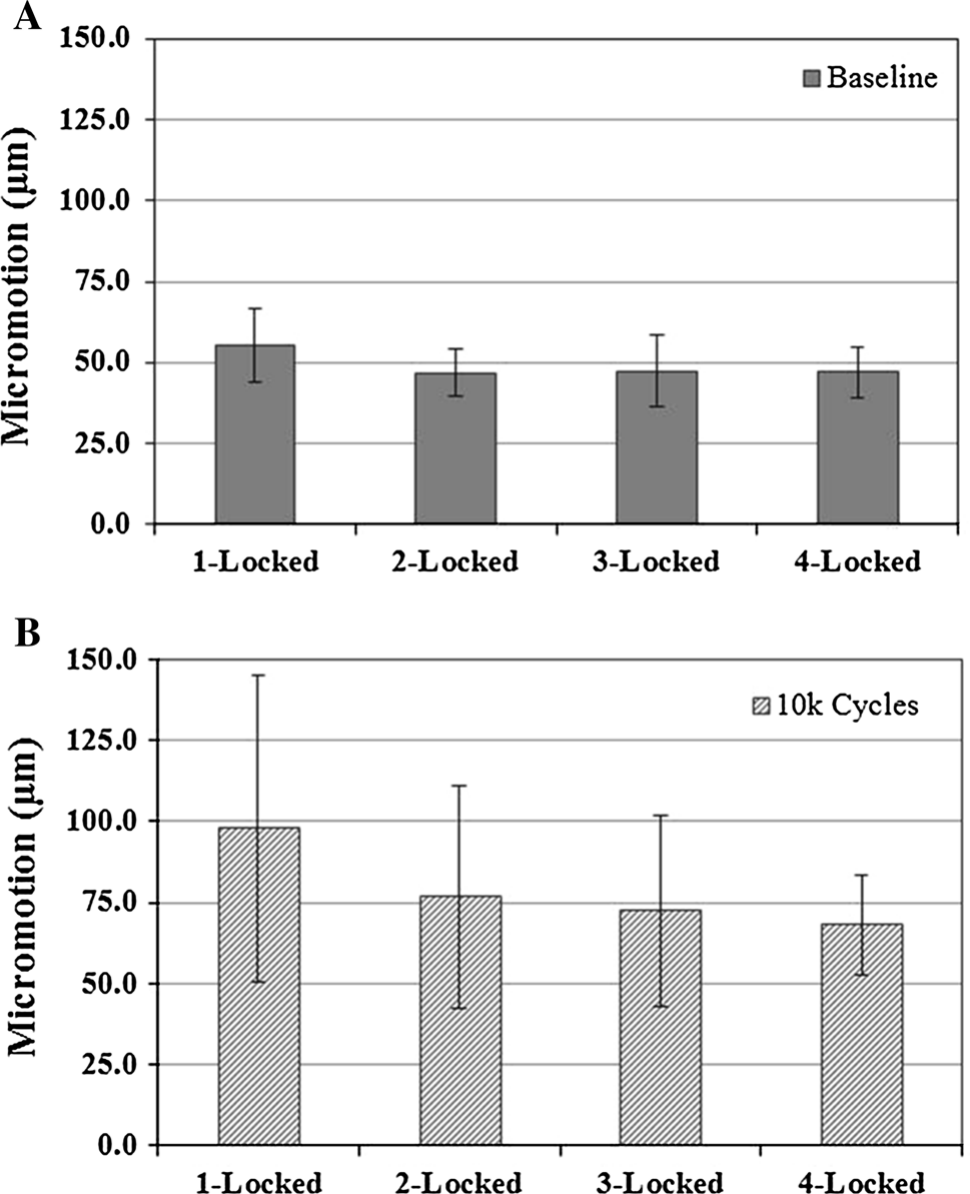
Mean and standard deviations of measured micromotion (μm) for each testing group at baseline (**a**) (*p* = 0.662) and after 10,000 cycles (**b**) (*p* = 0.695)

**Fig. 6:**
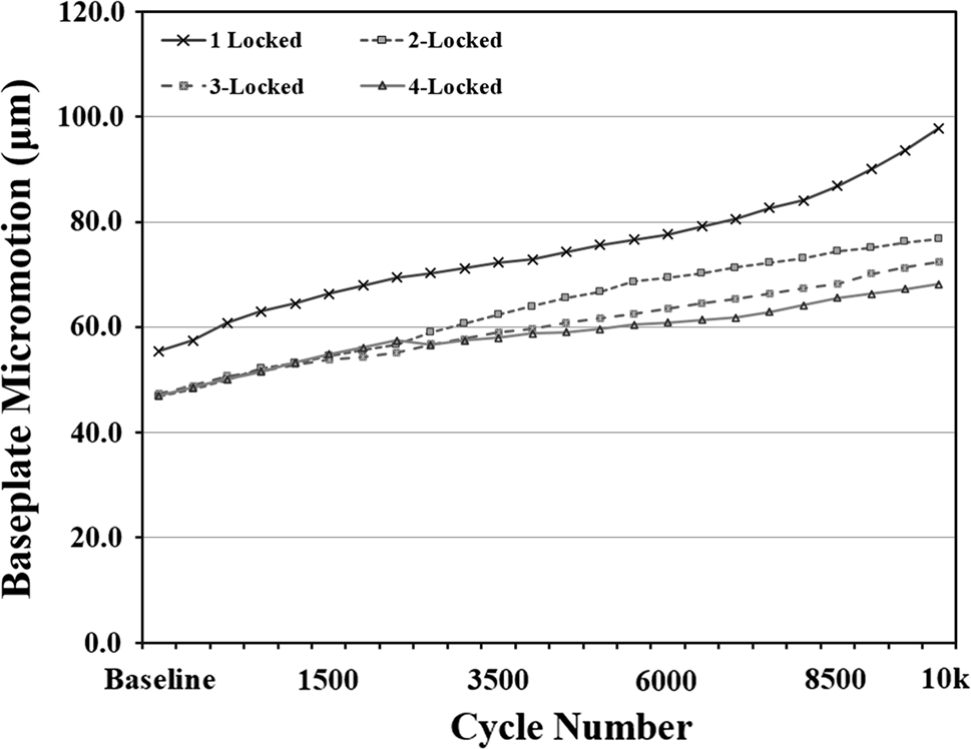
Temporal micromotion plot for all fixation groups

There was no difference in baseplate micromotion at baseline between any of the fixation groups (*p* > 0.662) (Fig. 5a). After 10,000 cycles of loading, the 4-locked fixation group exhibited the lowest measured baseplate micromotion, on average (68.1 ± 15.3 μm; range 45.2–91.0 μm), although this was not significantly different (*p* > 0.665) from the 1-locked (97.1 ± 47.2 μm; range 40.1–177.6 μm), 2-locked (76.7 ± 34.5 μm; range 43.1–143.1 μm), or 3-locked fixation groups (72.4 ± 15.3 μm; range 39.5–121.6 μm) (Figs. 5b, 6).

## Discussion

Critical to glenoid component survivability of the reverse shoulder replacement is obtaining sufficient initial fixation on the glenoid bone to facilitate osseous integration [4, 6, 11]. Minimization of micromotion <150 µm between an implant and bone has been shown to be optimal in achieving a biologic environment for bone growth onto metal arthroplasty surfaces [30–32]. Extrapolating these findings and applying them to the reverse shoulder glenoid component, numerous attempts have been made at enhancing baseplate fixation with the common goal of limiting micromotion between the glenoid component and the prepared glenoid surface. Over the past 15 years, advancements in surgical technique, implant design modifications, and understanding of scapular anatomy have helped surgeons improve baseplate failure rates. Of these enhancements, screw fixation has been noted as the most important feature of enhanced fixation [4, 9, 12].

Use of a glenosphere implant with a more lateral center of rotation was met with controversy based on historical implant failures seen with earlier generation implants [14–21]. The first modern reverse shoulder replacement to revisit a more lateralized center of rotation utilized a central screw baseplate and four peripheral non-locked 3.5-mm screws. Using all peripheral non-locked screws, the glenoid baseplate failure rate was 11% [22]. With continued understanding of surgical technique and replacement of the 3.5-mm peripheral non-locked screws with 5.0-mm locked screws, the incidence of glenoid baseplate failures was reduced to 0% in a 5-year minimum follow-up study [33]. This clinical experience validated the mechanical study reported by Harman et al. [11] which showed how peripheral locked screws were superior to non-locked screws at maximizing baseplate fixation <150 µm micromotion threshold. Surgeons have thus gained comfort using a glenoid baseplate with four peripheral locked screws. There are certainly cases where four peripheral locked screws are either not possible or may not be of optimal surgical preference. Included in these scenarios are cases where there is limited bone remaining, such that a screw would capture minimal bone or result in a prominent screw; or cases where a larger locked screw might not be optimal, creating a potential stress riser for an acromion spine fracture [34]. Additionally, there are situations when further glenoid component compression is desirable, which can only be achieved by using a non-locked screw. In these cases, the surgeon may elect to utilize a non-locked screw rather than a locked screw. This concept of hybrid fixation is now commonplace in fracture management using locked plates, but prior to this study had not been tested for applicability in the reverse shoulder replacement glenoid component.

Our study results demonstrate that a variety of hybrid peripheral screw combinations can provide sufficient glenoid component fixation using a glenosphere with a more lateral center of rotation. No statistical differences were observed in baseplate micromotion for peripheral screw combinations that included one, two, three and four peripheral locked screws. This was true at initial fixation (*p* > 0.662) and following 10,000 cycles (*p* > 0.665) using a bone substrate model that simulates poor glenoid bone quality often seen in reverse shoulder arthroplasty patients [13]. In fact, the maximum difference in measured micromotion between the extremes of groups (1-locked and 4-locked) was 29 µm (97 µm and 68 µm, respectively).

With increased cyclic loading of the glenosphere, there was increased micromotion seen at the baseplate−bone model interface. However, only one of the 28 tested constructs exhibited micromotion >150 µm threshold within the 10,000 cycles. This specimen utilized a single locked and three non-locked peripheral screws and did not reach this threshold until after 9500 cycles. While we noted no statistically significant differences between fixation groups, the variability in measured micromotion after 10,000 cycles of loading was decreased with an increasing number of locked peripheral screws. Thus, these study findings suggest that (1) hybrid fixation is a reasonable option for surgeons to consider when placing peripheral screws around a central screw baseplate and that (2) maximizing the number of peripheral locked screws, when clinically feasible, may more consistently maintain baseplate fixation through the postoperative rehabilitation course. Furthermore, use of a glenosphere with a center of rotation 10 mm lateral to the glenoid can be utilized with a glenoid baseplate secured with a combination of locked and non-locked screws without compromising the fixation necessary for ingrowth.

Several steps were taken to help maintain uniformity of the tested constructs and strengthen the validity of the data. Non-locked screws have the potential to lose fixation during implantation by stripping out. A pilot study was thus performed to evaluate the torque at which peripheral non-locked screws stripped (documented as a sharp drop in measured torque) within the bone model and lost fixation. All of the peripheral non-locked screws were then implanted within the torque limits observed, ensuring that each screw placed provided similar fixation. Additionally, the orientation of the baseplate was controlled such that the screw orientation remained constant, preventing variability in rigidity based on the orientation of the locked and non-locked screws. Finally, the central screw baseplates were implanted with an identical maximum torque to ensure that uniform compression was achieved with each tested model.

The cyclic loading protocol in this study mimics the stresses that might be observed in vivo and was similar to the technique utilized in our previous work [37] and by Roche et al. [29]. This consisted of a 750 N axial load applied through the humeral component while the glenosphere was rotated about the humeral component in a 55° arc of motion. This loading profile induces a maximum calculated shear force of 346 N and a maximum compressive load of 750 N. The applied compressive load magnitude of 750 N is in good agreement with data published by Bergmann et al. [35] who measured in vivo glenohumeral joint loads during abduction in patients with load/moment measuring total shoulder arthroplasty prostheses. However, the loading approach used here may be considered worst case, as muscle forces in reverse shoulder arthroplasty patients have been reported to vary between 30 and 50% less than in anatomical arthroplasty patients due to a compromised rotator cuff [36]. It was felt that this method of testing would better simulate what would be observed clinically rather than the methodologies noted by Harman et al. [11]. Nonetheless, the findings observed in the 4-locked screw group were similar to the findings reported by Harman et al. [11] using the identical 4-peripheral screw baseplate.

Limitations of this study relate to the use of polyurethane foam. While efforts were made to simulate the clinical environment, including placement of screws within torque limits, variations in patient bone quality may have different effects on hybrid peripheral screw baseplate fixation strength. Additionally, the screws placed were all within the foam model and did not obtain bi-cortical purchase that may occur in vivo. However, even without bi-cortical purchase, all but one non-locked baseplate configurations were able to limit micromotion <150 µm through 10,000 cycles. Finally, this study represents the initial fixation obtained by the glenoid baseplate. With the limitations of postoperative rehabilitation protocols, osseous integration of the glenoid baseplate may occur well before the 10,000 cycles tested in this study. Further investigation of the clinical outcomes using hybrid peripheral screw fixation of a central screw baseplate are warranted to validate the findings of this study.

In summary, hybrid peripheral screw fixation using combinations of locked and non-locked screws provides secure glenoid baseplate fixation using a polyurethane bone substitute model. Glenospheres with a more lateral center of rotation can be utilized together with a hybrid combination of peripheral glenoid baseplate screws without compromising fixation necessary for ingrowth of bone into the implant.
